# Interactions between Common Bean Viruses and Their Whitefly Vector

**DOI:** 10.3390/v16101567

**Published:** 2024-10-02

**Authors:** Amanda L. Ferreira, Murad Ghanim, Yi Xu, Patricia V. Pinheiro

**Affiliations:** 1 Institute of Tropical Pathology and Public Health (IPTSP), Universidade Federal de Goiás (UFG), Goiânia 74605-050, GO, Brazil; amandalopesferreira@discente.ufg.br; 2 Department of Entomology, Agricultural Research Organization, Volcani Center, Rishon LeZion 7505101, Israel; ghanim@volcani.agri.gov.il; 3 Department of Plant Pathology, Nanjing Agricultural University, Nanjing 210095, China; xuyiqdpd@njau.edu.cn; 4 Embrapa Arroz e Feijão, Santo Antônio de Goiás 75375-000, GO, Brazil

**Keywords:** *Bemisia tabaci*, *Phaseolus vulgaris*, insect–virus interaction, *Begomovirus*, *Carlavirus*, *Crinivirus*, *Cytorhabdovirus*

## Abstract

Common bean (*Phaseolus vulgaris* L.) is a widely cultivated crop, representing an important protein source in the human diet in developing countries. The production of this crop faces serious challenges, such as virus diseases transmitted by the whitefly *Bemisia tabaci*. Although there is a lot of information about some of these viruses, most of what we know has been developed using model systems, such as tomato plants and tomato yellow leaf curl virus (TYLCV). There is still very little information on the most relevant common bean viruses, such as bean golden mosaic virus (BGMV), bean golden yellow mosaic virus (BGYMV), bean dwarf mosaic virus (BDMV), cowpea mild mottle virus (CPMMV), and bean yellow disorder virus (BnYDV). In this review, we discuss the available data in the most up-to-date literature and suggest future research avenues to contribute to the development of management tools for preventing or reducing the damage caused by viruses in this important crop.

## 1. Introduction

Common beans, also known as dry beans (*Phaseolus vulgaris* L.), represent a widely cultivated crop, reaching an annual production of 28 mi t in 2022 [1]. India, Brazil, and Myanmar are the top three countries in dry bean production, area harvested, and consumption [1,2]. Mexico and Tanzania are the following countries in the dry bean consumption rank. Common bean grains have a high protein content and are also a rich source of carbohydrates, fibers, iron, and zinc [3]. It is not surprising that this legume represents the major source of protein in the human diet in developing countries and also an important component of their food culture [4]. Common beans are consumed daily by >500 million people worldwide [3,5] and are produced mainly by smallholder farmers in family agriculture systems. As additional benefits, the consumption of common beans has been associated with lower risks of cardiovascular diseases, diabetes, and obesity, as well as presenting an anti-inflammatory role [6,7,8,9].

Despite the importance of common beans as a healthy and relevant food source, the current average yield of the crop (~770 kg/ha) [1] is still below the potential of the most recent cultivars developed by the genetic breeding programs around the world. For example, some of the bean cultivars developed by Embrapa (Brazil) reach ~2500 kg/ha, with a yield potential of more than 4000 kg/ha, given the proper agronomic conditions [10]. Several factors influence the reduced yield potential of common bean cultivars around the world, including biotic stresses [11], mainly in tropical and subtropical regions, due to the combination of high temperature and air humidity. 

Common beans are highly susceptible to pests and diseases, including several viral diseases. In fact, common beans are, among the members of the *Leguminosae* family, the most susceptible species to virus diseases [10], especially plant viruses transmitted by the whitefly *Bemisia tabaci*. Depending on the cropping season and density of the whitefly population, yield losses can reach up to 100% [12,13]. Virus disease management is carried out mainly by chemical control of the insect vector population, with intensive use of pesticides. In some regions, when the vector population is very high, farmers carry out up to 15 insecticide sprays per cropping season [13]. In addition to being harmful to the environment and human health, the intensive use of insecticides increases the cost of production and the risk of selecting insecticide-resistant whitefly populations that can inflict more damage to common beans and other crops. Most insecticides used in agriculture are still fossil-based, contributing to an increase in the carbon footprint and undesirable impacts on the world climate. It is, therefore, urgent that new pest and disease management tools be developed.

The majority of plant viruses are spread by insect vectors, in complex biological systems that include several host plants, viruses, insect vectors, and their bacterial endosymbionts [14]. These biological players have co-evolved through intricate interactions over time: plant hosts activate immune defenses against viruses and insects, while insect vectors, viruses, and endosymbionts employ diverse strategies to evade, neutralize, or bypass these defenses [15,16,17], sometimes even in cooperation with each other [14,18,19]. As these biological entities interact, their biology and development are interdependent. Therefore, when describing one of them, it is often inevitable to mention details of the biology of the other players. However, virus management strategies usually consider only one of the players, mainly the host plant, without taking into account the role of the other actors in the interaction. Understanding the complex interactions between these actors is the first step to disrupting the interaction, which may lead to the development of a long-term and sustainable solution.

However, little is known about common bean viruses and their interaction with *B. tabaci*. Most of what we know was developed with model systems, usually with tomato and viruses from the same genus as those infecting common beans. However, there might be some subtle differences, due to the host plant and interactions with other players. For example, the combination of viruses in mixed infections in common beans may be from different families/genus from those that occur in tomatoes. These differences might influence the interaction between viruses and their insect vector. The objective of this review was to gather and discuss the information available in the literature about the most important whitefly-transmitted viruses that infect common beans, drawing attention to gaps and research possibilities, such as ways to interrupt the interaction between insect and virus, and not just between the virus and the plant, which is the case for the vast majority of studies carried out on these viruses.

## 2. Whitefly-Transmitted Viruses Infecting Common Beans

The whitefly *B. tabaci* is among the most important agricultural pests in the world. In fact, *B. tabaci* is considered a complex of biotypes or cryptic species containing at least 40 morphologically indistinguishable and reproductively isolated species [20]. These biotypes also differ in other adaptive traits, such as the ability to transmit plant viruses and the ability to develop resistance to insecticides [20]. The biotypes termed Middle-East Asia Minor (MEAM1) and Mediterranean (MED), also named B and Q biotypes, respectively, which seem to have originated in the Middle East, are global invasive pests. The most widespread biotype is MEAM1 [21]; however, in recent years, MED has spread, displacing MEAM1 and other indigenous species in several parts of the world, due to its resistance ability and tolerance to extreme conditions such as high temperatures. Several factors contribute to the success of the whitefly species complex members in colonizing a wide range of host species: its wide geographical distribution and presence in all continents; high efficiency in serving as vectors for plant viruses; highly extreme polyphagy, ability to colonize and feed on plants that belong to over 80 botanical families; high adaptability to adverse environment conditions; and rapid emergence of highly insecticide-resistant populations. Altogether, these traits place *B. tabaci* among the top 10 most invasive insect pests in the world [22]. Therefore, *B. tabaci* is among the insect pests that will likely benefit from climate change and global warming scenarios [23,24], which makes it an even more serious threat to food security, especially in developing countries. 

The most relevant economic damage caused by whiteflies is indirect, by the transmission of more than 400 plant viruses [25,26,27] that are responsible for significant crop losses. The majority of virus diseases that have become economically important in the last decades are transmitted by whiteflies [27]. Among the viruses transmitted by *B. tabaci*, the most important genus is *Begomovirus*, from the family *Geminiviridae*, which is transmitted exclusively by whiteflies. As an example, there are more than 60 begomoviruses transmitted by whiteflies to tomato plants worldwide. Due to its diversity, evolution, and worldwide spread, *B. tabaci* facilitates the emergence of new begomoviruses [28]. 

**Figure 1:**
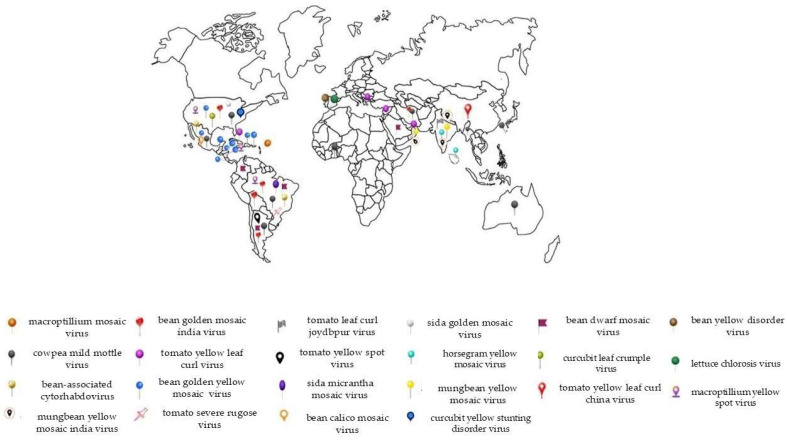
World map with markers representing different viruses transmitted by the whitefly (*Bemisia tabaci*) to the common bean (*Phaseolus vulgaris*). Each marker represents the geographic location where these viruses have been detected, with occurrences in various regions, indicating the widespread global distribution of these viruses. Viruses such as *Bean golden mosaic virus* (BGMV) and *Cowpea mild mottle virus* (CPMMV) are among the main contributors to significant losses in agricultural areas across Latin America, North America, Asia, and other regions. The recurrence of markers in numerous countries highlights the high dissemination potential of these viruses under various climatic conditions, driven largely by the wide distribution of their vector, the whitefly. The economic impact caused by these viral diseases varies by region, depending on the severity of the outbreaks and the management practices in place. This map provides a clearer view of the extent and the areas most affected by these different viral diseases. However, there are several gaps that should be addressed by research, for example, in countries like Myanmar, one of the largest bean producers, for which there is no information on the occurrence/frequency of viruses in beans.

In common beans, whitefly-transmitted viruses can cause yield losses that sometimes reach up to 100%, depending on the insect population and other climate factors. Our literature search returned 21 whitefly-transmitted viruses reported to infect common beans from the genera *Begomovirus*, *Carlavirus*, *Cytorhabdovirus*, and *Crinivirus* around the world (Table 1, Figure 1). Among these, only five are currently associated with economically relevant common bean diseases: bean golden mosaic virus (BGMV), bean golden yellow mosaic virus (BGYMV), bean dwarf mosaic virus (BDMV), cowpea mild mottle virus (CPMMV), and bean yellow disorder virus (BnYDV). For the remainder of the viruses listed, *P. vulgaris* is not the primary host, and therefore, the epidemiological relevance of these viruses to the crop is still low. However, considering the potential of virus outbreaks, it is important to review the virus species associated with beans and their interaction with their insect vector to be one step ahead in the development of management strategies aiming to prevent future losses. In the following subsections, we present the available literature on the biology of the interaction between insect vectors and viruses, comprising the members of four virus genera transmitted by the whitefly to common beans.

### 2.1. Begomoviruses

**Figure 2:**
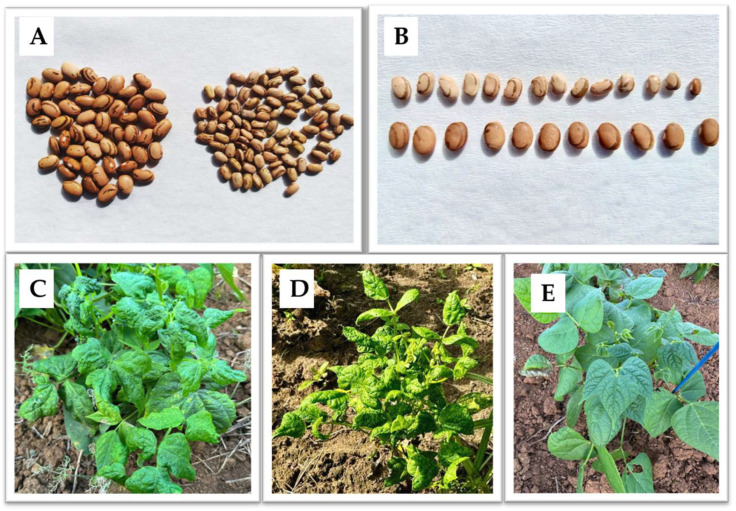
Virus symptoms in infected plants and seeds. (**A**,**B**): Seeds from cowpea mild mottle virus (CPMMV)-infected and uninfected common bean plants, highlighting the morphological differences caused by the viral infection. (**C**,**D**): Bean plants infected with bean golden mosaic virus (BGMV) (and other viruses in mixed infections), displaying severe symptoms caused by the viral infection. (**E**): Bean plant infected with CPMMV, displaying mild symptoms caused by the virus. (**A**,**B**) photos by Leandro Ribeiro de Matos, Embrapa Arroz e Feijão; (**C**–**E**) photos by Amanda Lopes Ferreira.

The genus *Begomovirus* is an important genus of the family *Geminiviridae*, which is characterized by twinned (geminate) particles and a single-stranded circular DNA genome (ssDNA). Most *begomoviruses* are exclusively transmitted by the whitefly *B. tabaci* and are not mechanically transmitted [29,30,31]. Some of these viruses can be mechanically transmitted, such as bean dwarf mosaic virus (BDMV), for example [32], and there is one report of another whitefly species transmitting TYLCV [33]. Some begomoviruses, such as BGMV, can cause highly severe symptoms in common beans, varying from yellow mosaic on leaves, leaf deformation, reduced leaf size, plant dwarfism, and a significant reduction in productivity (Table 1, Figure 2).

The mode of transmission of *begomoviruses* is persistent circulative. This means that after acquisition by the insect vector while feeding on an infected plant, virus particles pass through the mouthparts and esophagus to the midgut where they cross the gut epithelial cells to enter the hemolymph [34]. Extensive microscopy studies have shown that TYLCV localizes in the whitefly midgut, mainly in the filter chamber, where there is a higher concentration of virions in viruliferous insects, which suggests that the filter chamber is the main site of passage for TYLCV virions into the hemolymph [35,36,37,38,39,40]. From there, virus particles circulate in the hemolymph and reach the salivary glands, where they can be ejected along with saliva during feeding, being inoculated into the plant. Due to the virus circulation in the vector body, there is a latent period between acquisition and inoculation. For TYLCV, the minimum latent period was reported to be 21 h [41], but later, another study showed a shorter latent period of 8 h, ranging from 7 to 27 h, after acquisition in tomato [38]. The minimum latent period for other common bean-infecting begomoviruses has not been reported yet, to our knowledge. Although there is a latent period between acquisition and inoculation, the acquisition and inoculation access periods (AAP and IAP, respectively) are relatively short. For TYLCV, an AAP of 10 min and an IAP of 5.5 h were reported [39]. Similar AAP and shorter IAP values were reported for BDMV and horsegram yellow mosaic virus (HgYMV), being 20-30 and 15 min, respectively, for a transmission rate of 10% [32]. HgYMV was transmitted by a single whitefly with an efficiency of 36% [42]. The transmission efficiency of BDMV increased with longer AAP and IAP values, as well as with the number of insects [32].

Once acquired, most begomoviruses are retained in the whitefly vector for its entire life. One study suggested that TYLCV is not retained for the insect’s lifespan [41], but in another report, the authors state that when acquired after adult emergence, the virus is retained for the insect’s entire life [38]. Additionally, there are reports showing a shorter retention time, such as for TYLCSV and HgYMV [42,43]. For the other begomoviruses infecting common beans, this information has not been investigated yet, assuming that all begomoviruses are retained for the lifetime of the insect. Transovarial transmission has been demonstrated for some isolates of TYLCV, at different rates, but not for others [44,45,46]. Contradictory or inconsistent observations may be the result of the use of different protocols, intrinsic differences in the whitefly populations used in the assays, or between virus isolates [36]. Additionally, TYLCV and two other begomoviruses squash leaf curl virus (SCLV) and watermelon chlorotic stunt virus (WmCSV) were shown to be horizontally transmitted through mating between males and females of both *B. tabaci* MEAM1 and MED cryptic species [39,46,47]. Vertical and horizontal transmission of cucurbit leaf crumple virus (CuLCrV) has been reported, but at a low frequency, meaning that it is not epidemiologically relevant [48].

Begomoviruses have long been considered as nonpropagative in the insect vector; that is, the viruses cannot replicate in the insect tissues. However, most of what we know is based on a lack of evidence, instead on evidence that they cannot propagate in their vector. Indeed, virus replication in the *B. tabaci* MEAM1 has been demonstrated for TYLCV [49,50], suggesting that *B. tabaci* may be considered a host of this virus. In one of these works, viruliferous insects exposed to insecticides showed continuous virus accumulation, suggesting that the insect’s immune system might have compensated for the responses to the insecticide stress by indirectly impairing the response to TYLCV, leading to virus accumulation [50]. It is possible that, as other begomoviruses are studied in more depth, more evidence of replication in the insect vector will be reported. This result supports earlier research that demonstrated the virus’s detrimental effects on *B. tabaci* MEAM1 [51]. However, a different effect was reported for the MED cryptic species, in which TYLCV increased the insect’s performance [52]. Results reporting evidence for the lack of virus replication have been reported for BGMV and CuLCrV [48,53]. BGMV and BGYMV are the most important begomoviruses infecting beans in America [54,55]. Whereas BGYMV is the most prevalent in Central America and Mexico, BGMV occurs with a higher frequency in South America, predominantly in Brazil [30,54,56]. Despite their importance, there are relatively few studies on the interaction between these viruses and their insect vector, compared to TYLCV. For some biological aspects, the findings reported for TYLCV apply to the other begomoviruses. However, there are examples showing that it would be informative to carry out experiments with the specific virus, instead of using information developed for model systems.

It has been demonstrated, for example, that the transmission efficiency of begomoviruses varies among whitefly biotypes [57,58], which might be associated with the accumulation of virus particles in the insect after acquisition and with the virus efficiency to cross the insect’s midgut barriers [57,59,60,61]. The MEAM1 biotype of *B. tabaci* was more efficient in transmitting BGMV to common bean plants, compared to the MED biotype [60] and the native NW1 biotype [62]. However, the transmission efficiencies of BGMV by MEAM1 and NW2 biotypes were similar [62]. On the other hand, the MEAM1 biotype was less efficient than the biotype Asia II 1 in transmitting another *Begomovirus*, tobacco curly shoot virus (TbCSV) [57]. These variations show that some biological parameters cannot be generalized. Higher transmission efficiency rates of TYLCV were reported for whitefly populations with the presence or absence of the facultative endosymbionts from the genus *Hamiltonella* [63], or populations that differ in the prevalence of this endosymbiont [64]. This was also demonstrated for BGMV, when two populations of the MED species were compared, and the most efficient transmission was observed for the insect population with a higher prevalence of *Hamiltonella* [65].

Most of what is known about these pathosystems refers to the plant side of the interaction, addressing alternative hosts and the development of plant resistance. Some studies showed that alternative hosts serve as a reservoir for virus inoculum [66], which might lead to the development of new viruses through both recombination and pseudo-recombination events [67]. For example, a novel recombinant bipartite *Begomovirus*, named macroptilium bright yellow interveinal virus (MaBYIV), was reported as a recombination between BGMV and ToMoLCV, and it was found in mixed infections with BGMV in the weed *Macroptilium erythroloma* [67]. Indeed, weeds from the *Leguminosae* family, and most specifically the *Macroptilium* genus, have been reported as an important alternative host of these begomoviruses [67,68]. For some of these viruses, common beans are an alternative host, while other plants are better inoculum sources [69]. For example, the acquisition of sida golden mosaic virus (SiGMV) by whiteflies was higher from prickly sida than from *P. vulgaris* and other host plants [70].

Regarding the interaction between these viruses and the common bean plant, there are differences among domesticated and wild plant genotypes, related to virus prevalence and mixed infections, both conditions with a higher incidence in the domesticated genotypes [55]. A great effort has been made in the search for common bean resistant varieties to these viruses, or resistance/tolerance factors, and therefore, this is the most common subject of study in the literature about these pathosystems [71,72,73,74,75,76]. It is known, for example, that common bean varieties originated from the Andean gene pool usually exhibit a higher susceptibility to BDMV, while most varieties from the Middle American gene pool are resistant to this virus [76,77]. On the other hand, the majority of the common bean cultivars (domesticated) are highly susceptible to BGMV and BGYMV, although there is some variability in this trait. For example, the severity of BGMV symptoms varies with the common bean genotype and time after inoculation [78]. However, despite the search for sources of natural resistance to these two viruses [79], the only commercially available common bean cultivar with resistance to BGMV is a transgenic line with complete immunity to BGMV. Many research studies have addressed the development of this transgenic line [13,80,81,82,83,84,85,86,87,88]. Briefly, the genetic modification was developed using the RNA interference (RNAi) approach for silencing the virus *rep* gene, aiming to prevent virus replication in the plant. In addition to the absence of any virus symptoms, transgenic plants inoculated with BGMV did not bear any detectable virus DNA after the removal of the insect vector [83]. Additionally, it was shown that the DNA amount of the virus was significantly reduced in whiteflies feeding on the transgenic plants (compared with insects feeding on control non-transgenic plants), which suggests that BGMV might not be able to accumulate with high efficiency in the whitefly [53]. Later, another common bean transgenic line was developed, using the same RNAi approach to silence the vATPase gene of the whitefly. This time, the silencing conferred a moderate tolerance to the insect (about 60% insect mortality) [89].

### 2.2. Carlavirus

The genus *Carlavirus* is classified within the family *Betaflexiviridae*. The viruses in this genus are ssRNA positive-strand viruses, either transmitted by sap or by aphids, except for two viruses identified to date that are transmitted by whiteflies, CPMMV and melon yellowing-associated virus (MYaV) [90]. CPMMV infects plants in the *Leguminosae* family, such as common bean, soybean (*Glycine max*), peanuts (*Arachis hypogaea*), and cowpea (*Vigna unguiculata*). In soybean, CPMMV causes the stem necrosis disease, which is no longer considered a threat to the production of this crop, since most soybean cultivars are tolerant to CPMMV [91,92]. This means that soybean infected plants are symptomless, although virus replication still occurs. However, as soybean is one of the most cultivated crops in the world, it contributes to the continuous development of whitefly populations in the field and also serves as a relevant reservoir of CPMMV. Considering that there is a wide range of host plants and different CPMMV isolates reported around the world [93,94,95], symptoms in common bean plants can vary, from mild and asymptomatic infections (sometimes masked by the symptoms of begomoviruses in mixed infections) (Figure 2) [90,96,97,98,99,100] to highly severe symptoms and yield losses, which have been reported in the last several years, concerning farmers and researchers encountering more severe symptoms and yield losses [99].

Some isolates of CPMMV have been reported as seed-transmitted in soybean, cowpea, and yardlong bean. As there are only two viruses of this genus transmitted by whiteflies, identified relatively recently, there is less information about their interaction with the insect vector compared to begomoviruses [101]. Although it is known that CPMMV is transmitted in a non-circulative mode, whether it is semipersistent or nonpersistent is still controversial. In one study, an isolate of CPMMV from Thailand was reported as semipersistently transmitted [93], while in another work, an isolate from Ghana was reported as nonpersistent [94]. These differences may result from the use of different techniques for virus detection, but also due to genomic differences between virus isolates [101,102,103]. Conceptually, semipersistent viruses are retained in the vector tissues for days to weeks, attached to the foregut, while non-persistent viruses remain attached to the vector stylet for minutes to hours. CPMMV has been shown to be transmitted to soybeans after an acquisition access period (AAP) of 15 min and an inoculation access period (IAP) of 5 min [98]. However, virus persistence and localization in the insect have not been demonstrated yet [94,104,105], to our knowledge.

As a non-circulative virus, CPMMV is transmitted by the whitefly with less efficiency than begomoviruses, for example. A single whitefly can transmit CPMMV to 11.7% of test soybean or common bean plants [100], compared to 36% for the begomovirus HgYMV [42]. Transmission rates increased with the number of viruliferous insects used in the transmission assays, as well as with longer periods of virus acquisition and inoculation [98]. Interestingly, the MED species was more efficient in transmitting CPMMV when the inoculum and the test plant were from the same species (from common bean to common bean, for example) [106]. These authors also showed that the adult emergence of both whitefly cryptic species (MED and MEAM1) was higher on CPMMV-infected plants, compared to healthy plants [106], suggesting that virus acquisition conferred a fitness advantage to the insect vector.

The “vector manipulation hypothesis,” validated by many research groups, posits that pathogens are able to induce changes in the insect vector behavior or in the host plant they infect that indirectly influence the vector behavior, to enhance virus transmission [107,108,109,110,111,112,113,114,115,116,117,118]. These studies have shown that aphids are more attracted to plants infected with viruses that they transmit, compared to plants infected with other viruses or to healthy plants. Then, after a period of feeding on the virus-infected plant, viruliferous insects are more attracted to healthy plants, which contributes to virus dispersion. Several stimuli have been associated with an indirect manipulation of the virus on the vector behavior, such as alterations in the infected plant to produce volatile compounds and to change color, as a cue to attract the insects. A direct effect of the virus on the insects’ behavior has been demonstrated as well, when insects were fed on purified virus, and the same attraction to healthy plants was reported. The duration of feeding time on the infected plant should vary with the mode of virus transmission. For example, persistent-circulative viruses would benefit from longer feeding periods of the insect vectors, while stylet-borne viruses, which remain attached to the insect stylet for a short period of time, should move quickly to healthy plants [34].

Similar results have also been reported for whiteflies transmitting circulative and non-circulative viruses, showing that the manipulation hypothesis also applies to other species of insect vectors [119,120]. However, a different pattern was reported for CPMMV-viruliferous whiteflies, which were more attracted to infected plants, while non-viruliferous insects were more attracted to healthy plants [106]. This result indicates that the effect of virus acquisition on the insect vector performance and behavior is variable and so it should be investigated individually.

### 2.3. Cytorhabdovirus

The genus *Cytorhabdovirus* belongs to the family *Rhabdoviridae* and is characterized by having a negative-sense single-stranded RNA genome, ranging in length from 10 to 16 kb. These viruses are encapsulated in a bacilliform-shaped virion [121,122]. *Cytorhabdovirus* species accumulate in the cytoplasm [123] and are usually transmitted persistently and considered as propagative inside their insect vectors, predominantly hemipterans such as whiteflies, leafhoppers, and aphids, infecting both monocotyledonous and dicotyledonous plants from a wide range of families, including both weeds and major agricultural crops [123]. The symptoms of infection can vary significantly, ranging from stunting and vein clearing to leaf mosaic and tissue necrosis.

Several *Cytorhabdovirus* species illustrate the diversity of hosts and symptoms associated with these viruses. For example, barley yellow striate mosaic virus (BYSMV), transmitted to barley by planthoppers, causes characteristic mosaic patterns and discoloration; broccoli necrotic yellows virus (BNYV), spread by aphids, leads to severe yellowing and necrosis; and wheat American striate virus (WASMV), which affects wheat crops, is transmitted by leafhoppers and results in stunted growth and stripe-like lesions [124]. Recently, a new *Cytorhabdovirus*, named bean-associated *Cytorhabdovirus* (BaCV), was identified for the first time infecting common bean in Brazil [96]. This discovery represents the first description of a *Rhabdovirus* transmitted by the whitefly (MEAM1) [125], although the exact mode of transmission by the whitefly remains unknown. The most prominent symptom associated with this virus is leaf crinkling, which typically occurs in mixed infections with BGMV and/or CPMMV, complicating the visual identification of the virus [125].

The study of the geographical distribution of BaCV revealed its widespread presence across various regions of Brazil. The virus was detected in the states of Alagoas, Goiás, Minas Gerais, Mato Grosso, and São Paulo. The presence of BaCV in various bean-producing regions suggests that the virus has a broad geographic distribution and poses a potential risk to bean crops. BaCV has also been reported to naturally infect common beans in Mexico [55]. These findings underscore the importance of monitoring BaCV and conducting further studies to gain a more detailed understanding of the interaction between the pathogen and its host.

### 2.4. Crinivirus

*Crinivirus* is an emerging genus of the family *Closteroviridae*, whose members are semipersistently transmitted by whiteflies from the genera *Bemisia* and/or *Trialeurodes* [126]. They are considered emerging viruses because their whitefly vectors have been recently established in temperate climates worldwide [127]. Unlike other members of the *Closteroviridae* family, criniviruses have bipartite positive-sense ssRNA genomes. The symptoms induced by criniviruses (interveinal yellowing of leaves, leaf brittleness, reduced plant vigor, etc.) are often confused with physiological or nutritional disorders or pesticide-related phytotoxicity [126]. Interestingly, crinivirus symptoms are most prominent in mixed infections, whereas in single infections they are usually asymptomatic.

The model species (i.e., the most studied) of this genus is lettuce infectious yellows virus (LIYV). The capsid protein of LIYV has also been demonstrated to be required for whitefly transmission [128]. LIYV was retained in the whitefly for 3 days on susceptible hosts [129]. However, LIYV has not been reported infecting common beans yet. Bean yellow disorder virus (BnYDV) was the first crinivirus identified infecting plants of the *Leguminosae* family, first in Spain [130], and later in Tanzania [131] (Figure 1). After a BnYDV outbreak in Spain in 2004 and 2005, causing yield losses of 68% in greenhouse-produced green beans, another outbreak occurred in 2010, with similar symptoms. However, this new disease was associated with another crinivirus, lettuce chlorosis virus (LCV), which had been reported infecting lettuce plants in California, but not in Europe, and not in common beans. The new strain was named LCV-SP and it was further identified as a recombinant of BnYDV and LCV [130]. The transmission of BnYDV was more efficient after an AAP of 7 h and an IAP of 24 h, compared to 3 h and 12 h, respectively [132]. These authors also reported that the virus persisted in the MED species of *B. tabaci* for 2 weeks and that its host range was restricted to some plants in the *Leguminosae* family, excluding soybean, for example. 

In addition to these, another crinivirus was recently reported infecting *P. vulgaris*: cucurbit yellow stunting disorder virus (CYSDV) [133]. Although the epidemiological importance of CYSDV in beans is low, it is interesting to observe these “emerging” viruses infecting hosts outside their common range, which for CYSDV was mainly in the *Cucurbitaceae* family.

## 3. Molecular Interactions between Viruses and Insect Vectors

The biological aspects of the interaction between the whitefly and the viruses transmitted by this insect to the common bean plant differ among virus genera, as presented in Section 2. These differences are mainly due to different modes of interaction at the molecular level. For example, the primary difference among these groups of viruses is based on the mode of transmission by the insect, which can be either circulative or non-circulative in the insect vector. Both modes of transmission require specific molecular interactions, either for virus attachment in the insect stylet and other mouthparts, transport to the foregut, or crossing physical barriers in the other portions of the insect gut, hemolymph, and salivary glands. However, the molecular and cellular factors involved in the different modes of virus transmission by whiteflies are not yet well understood. Therefore, research pinpointing the key factors involved in their transmission is essential for developing strategies to interrupt the interaction, thus avoiding or reducing the efficient transmission of the virus. To our knowledge, there are no reports of omics studies on the molecular interactions between *B. tabaci* and common bean-infecting viruses, except for TYLCV, which is not associated with economically important diseases in common beans. However, studies carried out with TYLCV and other extensively studied viruses for non-circulative transmission by whiteflies serve as a first step in understanding these complex systems.

As a genetic inherited trait, vectoring competence varies among populations of an insect species [34]. This has been demonstrated mostly for insect vectors with specialist feeding habits, such as the aphid species *Schizaphis graminum*, which feeds only on plant species of the family *Poaceae* and is able to transmit multiple species of barley and cereal yellow dwarf viruses (B/CYDV). A series of studies showed that different populations of *S. graminum* differ in their ability to transmit plant viruses, in a continuum spectrum [134,135,136,137,138]. Vector and non-vector populations have the same feeding habits and ingest the same amount of virions from infected plants. However, the difference between these populations is related to the virus’s ability to circulate within the insect, pointing out that the insect–virus interaction is genetically controlled [34].

In contrast, little is known about the differences among insect populations regarding vector competence for generalist insects, which are usually highly efficient virus vectors. For example, to our knowledge, there are no reports of populations of the generalist aphid *Myzus persicae* with different levels of vector competence. For this insect species, vector competence was reduced when aphids were fed on a plant that is not a host of the virus, showing that the vectoring ability of this generalist aphid is indirectly influenced by the host plant [139]. For *B. tabaci*, also a generalist insect, differences in vectoring ability have been reported among cryptic species and among insect populations of the same species but with varying endosymbiont prevalence [140]. For generalist insects to feed on and establish themselves on a wide range of plant species, they must exhibit significant adaptability to counteract diverse plant defense systems. Therefore, feeding on different host plants may have a metabolic cost to generalist insects, which should be compensated by turning off other mechanisms, some of them related to vector competence. Natural selection has favored insect populations with the ability to compensate for their metabolic needs in order to feed on a wide range of host plants and to vector viruses, which may help to fight plant defenses. Although the regulations are distinct, these are also genetic regulated traits, triggered by environmental factors.

It has been known for a long time that the viral coat protein is essential for vector transmission and specificity [141,142,143,144]. However, although the whitefly genome has over 15 thousand protein-coding genes [22], little is known about insect proteins that interact with virus proteins. The whitefly GroEL protein was one of the first to be demonstrated interacting with a virus transmitted by this insect. Feeding the insects with an anti-GroEL antiserum prior to TYLCV acquisition significantly reduced virus transmission, suggesting that this protein protects the virus particles during their circulation through the hemolymph [145]. Later, other studies showed that the GroEL produced by the endosymbiont *Hamiltonella* specifically interacts with the TYLCV CP [63] and this interaction occurs in the cryptic species MEAM1, which is an efficient vector of TYLCV, but not in the MED species, a poor vector of this virus. Interestingly, while the GroEL produced by *Hamiltonella* facilitates virus transmission, the other endosymbionts do not seem to contribute to the insect’s vector competence. Another whitefly protein reported to be involved in the transmission of TYLCV was heat shock protein 70 (HSP70), which was upregulated in response to acquisition of TYLCV and SLCV, interacted with TYLCV in vitro, colocalized with this virus in the midgut, and presented an inhibitory effect on virus transmission [146,147].

As access to high-throughput molecular research techniques becomes available and less expensive, several studies have been carried out to look for genes that are differentially expressed in *B. tabaci* after virus acquisition as well as proteins associated with different steps of the interaction, such as virus entry in the insect cells, movement through insect tissues, or even skipping plant or insect defenses [148,149]. These studies show that the acquisition of plant viruses (or virus infection in the plant) is perceived by the insect in a specific way, being different for each species of virus and even for each cryptic species of *B. tabaci* [146,149]. Additionally, these studies generally report a set of genes that are differentially expressed in viruliferous insects compared to nonviruliferous, that is, associated with virus acquisition, regardless of the virus species or mode of transmission [146,150].

In general, TYLCV and TYLCCNV acquisition by whiteflies resulted in differential expression of genes involved in the cell cycle, primary metabolism, and immune responses, among other pathways [146,151,152]. These results contradict the idea that whitefly-transmitted viruses pass unnoticed by the insect’s organs and defenses. One group of genes that was found to be differentially expressed in both MEAM1 and MED cryptic species of *B. tabaci* after acquisition of two viruses transmitted in different modes (TYLCV and ToCV) was the cathepsin family, including cathepsin-B, cathepsin L-like, cathepsin F, and cathepsin F-like genes [22,146,152]. In general, cathepsin genes were upregulated in TYLCV-viruliferous whiteflies and downregulated in ToCV-viruliferous whiteflies [22,146]. Interestingly, the genome of *B. tabaci* MEAM1 presents several copies of cathepsin genes, representing a significant expansion in comparison with those observed for other arthropods. For example, there are 50 copies of cathepsin-B genes and 35 of cathepsin L-like [22], which could be linked to the insect’s exceptional efficiency as a vector for a wide range of plant viruses. An increased activity of cathepsin-B and other cysteine proteases at the cell membrane had been reported for the aphid *M. persicae,* which indirectly decreased the transmission of the *Luteovirus* PLRV by this insect [139]. Other genes that showed relevant differential expression after TYLCV acquisition by the whitefly were hemocyanin, orexin, a lipoprotein receptor known to be involved in receptor-mediated endocytosis of viruses [152], and cytochrome P450 [146]. Differential gene expression has also been reported when comparing different AAPs, suggesting that the insect response is time-dependent, as is virus accumulation in the insect, which increases with longer AAPs [150]. Although several studies on the transcriptional response of *B. tabaci* to TYLCV acquisition have been carried out, the results are not always similar. For example, differentially expressed genes considered relevant in some studies, such as genes from the cathepsin family [22,146,152], were not reported in other studies [150,151,153], showing that the results of transcriptomic studies vary in relation to the protocols used and other experimental conditions.

**Table 1:** Common bean (*Phaseolus vulgaris*) viruses transmitted by the whitefly *Bemisia tabaci*.

Virus Family, Genus	Virus Name	Countries Where They Have Been Reported	Symptoms	Severity	References
*Geminiviridae*, *Begomovirus* is a genus that consists of twinned (geminate) particles with a single-stranded circular DNA genome (ssDNA) Virus species model: TYLCV in tomato Transmission mode: circulative, persistent, and nonpropagative (?) Highly efficient transmission (100% plants infected with 5–15 insects) Reviewed in [28]	tomato yellow leaf curl virus (TYLCV)	Cuba, Greece, Oman, Syria, Israel, China, Brazil	Yellow mosaic and/or leaf crumple	The severity of TYLCV in bean crops can vary depending on the region. In most regions, the severity is low	[154,155,156,157,158]
	bean golden mosaic virus (BGMV)	Brazil, Bolivia, Argentina, USA, Iran	Yellow mosaic on leaves, leaf deformation, reduced leaf size, plant dwarfism, significant reduction in productivity	High severity, causing up to 100% yield loss in cases of early infection	[66,159,160]
	bean Golden Yellow Mosaic Virus (BGYMV)	Dominican Republic, Guatemala, El Salvador, Haiti, Honduras, Costa Rica, Mexico, USA, Nicaragua, Iran	Intense yellowing, pod deformation, stunting, and flower abortion	High yield losses to common beans grown in tropical and sub-tropical countries of Latin America and the Caribbean	[10,71,159]
	macroptilium yellow spot virus (MaYSV)	Brazil, Jamaica, USA	Chlorotic spots on the soybean leaves	Not reported	[161,162,163]
	tomato leaf curl Joydebpur virus (ToLCJoV)	India	Curling, yellow mosaic, and stunting	High severity	[164]
	sida micrantha mosaic virus (SimMV)	Brazil	Golden mosaic, chlorotic spots, and leaf distortion	Low severity	[165]
	sida golden mosaic virus (SiGMV)	USA	Foliar mottling, puckering, and curl	High severity, particularly in the Southeastern United States	[70,166]
	horsegram yellow mosaic virus (HgYMV)	Sri Lanka, India	Included a bright yellow mosaic pattern on the leaves, rugosity, reduced leaf size, and stunting of the entire plant	High severity, depending on the timing of infection, plants produce fewer flowers and pods or none at all	[167,168]
	mungbean yellow mosaic virus (MBYMV)	India, Thailand	Yellow mosaic, puckering, and a reduction in size	High severity. Early-infected plants typically die without forming pods	[31,169]
	mungbean yellow mosaic India virus (MBYMIV)	India, Oman, Nepal	Yellowing of the veins and leaf crumpling	Low severity	[170,171]
	bean dwarf mosaic virus (BDMV)	Brazil, Argentina, Colombia, Saudi Arabia	Severe dwarfing, leaf distortion, mottling or mosaic, and chlorotic spots	High severity if infection occurs early	[10,32,66,172,173,174]
	cucurbit leaf crumple virus (CuLCrV)	USA	Leaf deformation, rugosity, and mild mosaic	Low severity	[175]
	tomato yellow leaf curl China virus (TYLCCNV)	China	Leaf curl	Low severity	[176]
	macroptilium mosaic virus (MaMV)	Puerto Rico	Green-yellow mosaic foliar symptoms and stunting	Low severity	[177]
	tomato yellow spot virus (ToYSV)	Argentina	Yellow spots, mosaic, chlorosis, stunting, leaf deformation	Low severity	[178]
	bean calico mosaic virus (BCMoV)	Mexico	Yellow or white spots, chlorosis, leaf deformation, and stunted growth	Low severity	[179]
	tomato severe rugose virus (ToSRV)	Brazil	Asymptomatic	Low severity	[180]
*Betaflexiviridae, Carlavirus*	cowpea mild mottle virus (CPMMV)	Taiwan, Brazil, Australia, Argentina, USA, Mexico, Ghana, Thailand, Iran	Vein chlorosis, mild mottling, and leaf roughness	High severity	[66,93,94,102,181,182,183,184]
*Rhabdoviridae, Cytorhabdovirus*,	bean-associated cytorhabdovirus (BaCV)	Brazil, Mexico	mosaic, leaf distortion, crumpling, and dwarfing	Low severity	[96,125]
*Crinivirus*	bean yellow disorder virus (BnYDV)	Spain, Tanzania	Interveinal spots and leaf yellowing	Moderate severity	[131,132]
	lettuce chlorosis virus (LCV-SP)	Spain	Internerval mottling and yellowing on middle and lower leaves	Low severity	[185]
	cucurbit yellow stunting disorder virus (CYSD)	USA	Severe stunting and desiccation of leaves	Low severity	[133]

Similarly to the whitefly response to circulative viruses, the acquisition of a non-circulative virus (ToCV) also resulted in the differential expression of genes, compared to nonviruliferous whiteflies [148]. However, the number of differentially expressed genes (DEGs) was lower in ToCV-viruliferous insects, compared to insects that acquired the *Begomovirus* TYLCV [152]. When comparing different AAPs, the number of DEGs was higher after a 24 h AAP, both in the comparison between ToCV-viruliferous and non-viruliferous insects, and in the comparison between ToCV and TYLCV [148,152]. Other studies showed three large clusters of *B. tabaci*-specific genes, with undefined functions, and immune response-related genes differentially expressed in ToCV-viruliferous whiteflies [22,146]. The number of immune response genes differentially expressed in ToCV-viruliferous whiteflies was higher than in TYLCV-whiteflies or in the co-infection, suggesting that acquisition of a semipersistent virus triggers a stronger immune response in the insect [146].

To our knowledge, there is still no research on the molecular factors involved in the transmission by whiteflies of the most relevant common bean viruses (BGMV, CPMMV, BGYMV, BDMV, and BnYDV). It is not known which proteins would be involved, or whether the same proteins identified for TYLCV would be associated with the transmission of common bean viruses, but these interactions are probably specific and should be studied on a case-by-case basis. As high-throughput sequencing technologies become increasingly accessible for transcriptomics studies, investigating the differential expression of genes in the insect after acquisition of common bean viruses, or in the plant after virus inoculation, may be a first step to search for strategies to interrupt the virus–insect interaction, including via plant genetic modification, in the future.

## 4. Future Perspectives

Several viruses infect the common bean plant, causing diseases that impact productivity. In recent years, reports have emerged about the discovery of novel viruses infecting beans, such as BaCV, or viruses that were commonly found infecting other crops. The majority of these new viruses are not yet economically relevant. However, there is the imminent possibility of outbreaks, for which research must be prepared, given the importance of common beans as a food and as part of people’s cultures. Furthermore, there is a lack of information on the occurrence of viruses infecting beans for some regions, especially African countries, and one of the major bean producers in Asia, Myanmar. Surveys of the occurrence and diversity of viruses in beans and cryptic whitefly species in these regions are important for the development of integrated pest/disease management strategies.

Considering that the majority of research carried out with bean viruses is for the development of virus-resistant/tolerant plant lines, carried out by various groups around the world, the research field of insect–virus interaction is proportionally poorly represented, starting from understanding the basic vector biology and virus–vector interaction, up to the interactions at the molecular levels which could produce valuable results for future research directions. Additionally, knowledge of the specific relationships between bean viruses and the cryptic whitefly species predominant in each region is essential for the selection of genes/pathways that should be used as targets for research aimed at developing resistant plant lines, for example, selecting insect or virus genes to be used as targets for gene silencing using RNA interference, either by plant transformation or dsRNA delivered as a pesticide. Research carried out with model systems should serve as guidance for future studies that consider the specificities of each system. Furthermore, as most of these viruses occur in mixed infections, it is necessary to know their distribution frequency and their transmission dynamics in mixed infections, with regard to the transmission parameters such as AAPs, IAPs, retention time, and more.

Considering the threat of climate change, global warming, and the negative impacts of the use of fossil-based insecticides in agriculture, the rational management of pesticides must be considered a priority. The whitefly is a hazard to the production of food and other agricultural items not only due to its high capacity to transmit viruses but also due to the rapid development of insect populations resistant to insecticides. There is at least one study showing that viruliferous insects under insecticide stress accumulate a higher viral load, indicating possible replication of the virus in the insect. Future research should verify how insect populations interact with viruses in the field, under conditions of high pressure from insecticide spraying, as occurs in tropical countries.
